# A cost-effectiveness modeling study of treatment interventions for stage I to III esophageal squamous cell carcinoma

**DOI:** 10.1186/s12962-022-00352-5

**Published:** 2022-04-02

**Authors:** Rajabali Daroudi, Azin Nahvijou, Mohammad Arab, Ahmad Faramarzi, Bita Kalaghchi, Ali Akbari Sari, Javad Javan-Noughabi

**Affiliations:** 1 Department of Health Management and Economics, School of Public Health, Tehran University of Medical Sciences, Tehran, Iran; 2 Cancer Research Center, Cancer Institute of Iran, Tehran University of Medical Sciences, Tehran, Iran; 3 Department of Health Management and Economics, School of Public Health, Urmia University of Medical Sciences, Urmia, Iran; 4 Radiation Oncology Research Center, Tehran, Iran; 5 Social Determinants of Health Research Center, Mashhad University of Medical Sciences, Mashhad, Iran

**Keywords:** Esophageal cancer, Stage, Cost-effectiveness analysis

## Abstract

**Background:**

Esophageal cancer causes considerable costs for health systems. Appropriate treatment options for patients with esophageal squamous cell carcinoma (ESCC) can reduce medical costs and provide more improved outcomes for health systems and patients. This study evaluates the cost-effectiveness of treatment interventions for patients with ESCC according to the Iranian health system.

**Material and methods:**

A five-state Markov model with a 15-year time horizon was performed to evaluate the cost-effectiveness of treatment interventions based on stage for ESCC patients. Costs ($US 2021) and outcomes were calculated from the Iranian health system, with a discount rate of 3%. One-way sensitivity analyses were performed to assess the potential effects of uncertain variables on the model results.

**Results:**

In stage I, the Endoscopic Mucosal Resection (EMR) treatment yielded the lowest total costs and highest total QALY for a total of $1473 per QALY, making it the dominant strategy compared with esophagectomy and EMR followed by ablation. In stages II and III, chemoradiotherapy (CRT) followed by surgery dominated esophagectomy. CRT followed by surgery was also cost-effective with an incremental cost-effectiveness ratio (ICER) of $2172.8 per QALY compared to CRT.

**Conclusion:**

From the Iranian health system’s perspective, EMR was the dominant strategy versus esophagectomy and EMR followed by ablation for ESCC patients in stage I. The CRT followed by surgery was a cost-effective intervention compared to CRT and esophagectomy in stages II and III.

**Supplementary Information:**

The online version contains supplementary material available at 10.1186/s12962-022-00352-5.

## Introduction

According to the GLOBOCAN estimates, esophageal cancer (EC) is the seventh most common cancer type globally. Iran has been a high-incidence area of EC for many years and is located on the esophageal cancer belt. The EC incidence rate was 2.53 per 100,000 population and 5.3 per 100,000 population in 2001 and 2018, respectively [1, 2]. In developing countries, esophageal squamous cell carcinoma (ESCC) is the most common subtype of EC. For example, more than 64% of EC patients are diagnosed with ESCC in Iran [3, 4].

Generally, appropriate treatment interventions for patients with ESCC depend on variables such as cancer histology, age of patients, comorbidities, and stage detecting. In the initial stage, curers are usually adopted Endoscopic Mucosal Resection (EMR), esophagectomy, and EMR followed by ablation. In the middle stage (II and III), esophagectomy, CRT followed by surgery or CRT alone is applied [5].

Overall, physicians face challenges in using a suitable intervention for patients with EC. Esophagectomy is the most commonly used treatment for EC patients, although it is accompanied by considerable mortality and morbidity [6, 7]. Studies reported that after esophagectomy, the postoperative mortality rate was 1% to 11%, and postoperative complications were reported at 11% to 35% [8, 9]. In contrast, EMR and CRT followed by surgery are recommended to treat ESCC patients as an alternative to esophagectomy. Studies showed that after the treatment with EMR, approximately 3% to 6% of patients would incur a complication, with about a 17% to 28% complication rate seen in chemoradiotherapy followed by surgery [10, 11]. Also, studies reported death rates of 2 to 5% for CRT but did report any death rates for the use of EMR [12–14]. Additionally, the treatment cost is a substantial factor in the use of therapeutic interventions by patients. Thus comparing the effectiveness and the treatment cost on therapeutic procedurals may be helpful in selecting competing strategies, especially under conditions of uncertainty [15]. Cost-effectiveness analysis (CEA) is a practical tool to evaluate therapeutic interventions based on costs and outcomes. In this analysis, consequences are presented as a unit, such as cases of a disease prevented and years of life gained— with results inferenced in terms of the incremental cost-effectiveness ratio (ICER) [16].

Most studies on economic evaluation analysis associated with EC have been conducted to screen and treat Barret’s esophagus [17–19]. Besides, most CEA studies related to the treatment interventions on EC have been performed on the subtype of adenocarcinoma, most of which were conducted in developed countries [20–23]. We believe that the curing of ESCC patients based on the disease stage can reduce the cost of treatment and provide improved results for health systems and patients. Therefore, we designed a study to evaluate the cost-effectiveness of treatment interventions for patients with ESCC, based on the disease stage in Iran.

## Methods

To help decision-makers, in identifying appropriate treatment options for their patients, we performed a CEA to evaluate the costs and effectiveness of therapeutic procedures, based on disease stage for patients with ESCC.

We used the ICER, the ratio of the incremental cost to the incremental benefit of two competing interventions, for our analysis. The thresholds that WHO defined for low-income and middle-income countries were used. This threshold is one to three-times the gross domestic product (GDP) per capita [24]. The one to three-times GDP per capita for Iran is US$ 5627 to $16,881 [25]. We conducted deterministic sensitivity analyses to evaluate the potential effects of uncertain variables on the model results. For this reason, one-way sensitivity analyses were applied. We obtained the value for possible variables, using the maximum and minimum values for all variables extracted from the literature and medical records.

### Patients and interventions

We considered patients 60-years-old with stage I to III ESCC who received a treatment intervention as the target population because the studies displayed that most patients with esophageal cancer develop the disease in the aged 50 to 70 years [12, 26, 27]. Also, the medical records at the cancer institute of Iran demonstrated that the average age of ESCC patients was about 60-years. Patients were excluded if they were in the IV stage or high-grade dysplasia, as well as patients who had adenocarcinoma esophageal cancer. Also, we excluded patients that had other comorbid cancers simultaneously, such as gastric cancer, gastroesophageal junction cancer, oral cancer, Barrett’s esophagus, and other cancers. Patients were simulated based on the disease stage and followed until 75 years or death. We used the National Comprehensive Cancer Network (NCCN) guidelines and expert opinions to classify patients and identify interventions [28, 29]; therefore, the patients were identified stage I if they were graded as T1N0M0. Also, patients at stages II and III were graded as T2-4N0-2M0. Furthermore, we selected interventions of esophagectomy, EMR, and EMR followed by ablation for stage I patients. Esophagectomy, CRT, and CRT followed by surgery were chosen for stages II and III [29]. In the esophagectomy group, surgery was performed open. The esophageal resection among esophageal cancer patients could be performed in an open or minimally invasive manner. The minimally invasive technique causes fewer side effects in some variables (intraoperative blood loss, in-hospital mortality, and cardiovascular complication) [30]. However, open esophagectomy is most common [31, 32]. In the CRT group, the drug regimens were Cisplatin and Fluorouracil or Cisplatin with Docetaxel, with a total radiation dose of 5000 Gy was given in 25 fractions. The period of treatment was 4 to 6 months [28].

### Model design and assumptions

**Fig. 1:**
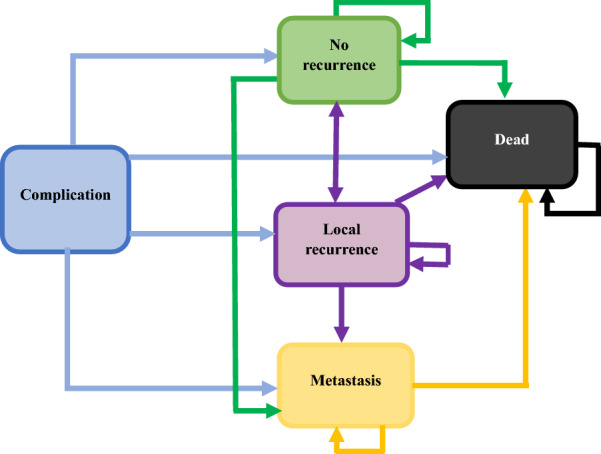
Markov model

Using tree age software (Tree age, Williamstown, MA, 2018), we employed a Markov model to perform a CEA of treatment interventions for patients with stage I to III of ESCC, based on disease stage. We adopted the Markov model for this cost-effectiveness study because the patients with esophageal cancer could be exposed to different health states after receiving therapeutic interventions during the natural history of the disease. In designing the Markov model, we conducted a systematic review associated with the economic evaluation of EC treatments. This systematic review is explained elsewhere [23]. Based on the results of the systematic review, we created a 5-state Markov model. Health states in the Markov model included: no-recurrence, local recurrence, metastasis, complication, and dead. The Markov model evaluated the outcomes over a 15-year time horizon (after which > 95% of patients had died in all interventions), using a 6-month cycle length. Figure 1 shows the Markov model and the transitions between the health state for each treatment. We assumed the initial probability at zero (for treatment) for the metastasis and local recurrence state in the Markov modeling. Patients then entered these states via the transition probabilities in the following cycles. The initial and transition probabilities for every treatment modality were extracted from previously published literature and then adopted for the 6-month cycles. Table 1 displays the transition probability for treatment interventions.

**Table 1:** Model input estimates

Description	Base case	Range	Reference
Stage I			
Esophagectomy			
Complication	0.119	0.119–0.35	[33, 34]
Dead	0.055	0.018–0.111	[8, 34]
No recurrence to local recurrence	0.0239	–	[35]
No recurrence to metastasis	0.0139	0.002–0.0139	[35–37]
No recurrence to Dead	0.07		[38]
EMR			
Complication	0.069	0.030–0.069	[6, 10, 12]
Dead	0	–	[12]
No recurrence to local recurrence	0.0188	0.0076–0.0426	[6, 12, 37, 39]
No recurrence to metastasis	0.0233	–	[37]
No recurrence to dead	0.07	–	[12, 37, 38]
EMR followed by ablation			
Complication	0.12	0.068–0.222	[40, 41]
Dead	0		[42]
No recurrence to local recurrence	0.0287	0.014–0.033	[43–45]
No recurrence to metastasis	0.0151	–	[45]
No recurrence to dead	0.082	–	[46]
Stage II and III			
Esophagectomy			
Complication	0.25	0.25–0.39	[35, 47]
Dead	0.06	0.0339–0.111	[8, 48]
No recurrence to local recurrence	0.1085	–	[49]
No recurrence to metastasis	0.0309	–	[49]
No recurrence to dead	0.085	0–0.0905	[47, 49, 50]
CRT followed by surgery			
Complication	0.2	0.17–0.289	[11, 51]
Dead	0.06	0.04–0.105	[5, 52]
No recurrence to local recurrence	0.0151	0.003–0.0151	[38, 53]
No recurrence to Metastasis	0.0123	0.0073–0.0123	[53, 54]
No recurrence to Dead	0.08	–	[52, 53]
CRT			
Complication	0.17	0.148–0.28	[13, 14]
Dead	0.04	0.024–0.058	[13, 14]
No recurrence to local recurrence	0.0528	0.028–0.0528	[14, 55]
No recurrence to metastasis	0.0139	0.0221–0.0139	[14, 55]
No recurrence to dead	0.13	–	[6, 13, 56]
COST ($ US)			
Esophagectomy	3464	–	Health system Iran
EMR	1788	–	Health system Iran
EMR followed by ablation	2136	–	Health system Iran
CRT followed by surgery	4762	–	Health system Iran
CRT	2410	–	Health system Iran
Utility			
No esophageal cancer	1	–	Assumption
Stage I esophageal cancer	0.693	0.66–0.71	[57, 58]
Stage II and III esophageal cancer	0.76	0.74–0.78	[57, 58]
Stage IV esophageal cancer	0.75	0.73–0.77	(57, 58)
Death	1	–	Assumpation

### Costs and effectiveness outcomes

We used the Iranian health care system as the perspective to estimate the costs. To calculate intervention costs, we used the medical records of ESCC patients at Iran's cancer institute in 2018. For this purpose, we identified direct costs, including direct medical costs and direct non-medical costs. The direct medical costs were considered for diagnosis cost, treatment, follow-up, and terminal care. The unit cost is defined for diagnostic tests, follow-up care, and treatment modalities from the Iranian health system perspective. The diagnosis costs are considered for services such as endoscopy, biopsies, endoscopic ultrasound, complete blood count (CBC), blood urea nitrogen (BUN), creatinine, serum glutamic oxaloacetic transaminase (SGOT), serum glutamate pyruvate transaminase (SGPT), alkaline phosphates, fasting blood sugar (FBS), and computed tomography (CT) scan. The follow-up cost includes visits, counseling, and CT scan. Direct non-medical cost refers to a proportion of out-of-pocket payment that must be paid by the patient [59]. Since the data and studies about the direct non-medical costs associated with esophageal cancer patients were lacking, we estimated only the traveling costs. In this regard, the number of clinical visits was extracted from medical records, assuming it was equal to the number of outpatient visit days. Then the cost of a trip was calculated based on expert' opinion. All costs were adjusted to $US 2021. The costs and outcomes were discounted at 3% [60, 61].

The primary outcome was life-years gained (LYG) and quality-adjusted life-years (QALYs). We calculated the QALY for the treatment interventions using the utility weights in the various health states. Utility weights were derived from the published literature [57, 58]. As in previous studies, we assumed a perfect quality of life (QOL = 1) for people with non-esophageal cancer [62, 63]. Other outcomes, such as complications (adverse events that occur as a direct result of the treatment used), postoperative mortality, and transition probability associated with the baseline health state, were derived from the literature (See Table 1 below for references).

## Results

### Base case results

In the base case analysis, the use of EMR yielded the lowest total costs ($4485), and highest total life-years gained (4.363) and QALY (3.045), making it the dominant strategy in stage I. Table 2 shows the CEA results for the base case model in patients with ESCC.

**Table 2:** Cost-effectiveness analysis of base-case for patients with ESCC

Treatment interventions	Total cost per patient (US $)	QALY	LYG	ICER (US$/QALY)	ICER (US$/LYG)
Stage I					
EMR	4485.6	3.045	4.363	Reference	Reference
Esophagectomy	5582.6	3.033	4.353	Dominated	Dominated
EMR followed by ablation	4753.9	2.884	4.136	Dominated	Dominated
Stage II and III					
CRT	4738.9	2.143	2.821	Reference	Reference
CRT followed by surgery	6707	3.048	4.013	2172.8	1650.9
Esophagectomy	7622.8	2.664	3.509	Dominated	Dominated

For stage II and III on ESCC, the CRT followed by surgery strategy resulted in 3.048 QALYs, while the CRT intervention yielded 2.143 QALYs and esophagectomy 2.664 QALYs. The total costs for the patient with ESCC were $4738 for CRT treatment, $6707 on CRT followed by surgery, and $7622 at esophagectomy. CRT followed by surgery dominated esophagectomy and; compared to CRT was cost-effective with an ICER of $2172 per QALY.

### Sensitivity analysis

The one-way sensitivity analysis can be found in Table 3. There was no change in the base case analysis for the results on stage I, and EMR was a dominant strategy. If the probability of death for the esophagectomy intervention was reduced by 0.041, the ICER of esophagectomy versus EMR would be $34,768 per QALY. The ICER would be $24,377 per QALY if the transition probability of no-recurrence to the metastasis state was reduced from 0.0139 to 0.0109 for esophagectomy. In stages II and III of ESCC, the results were sensitive to transition no-recurrence to the dead state in esophagectomy. By reducing this probability from 0.085 to 0.0452, esophagectomy was cost-effective compared to CRT followed by surgery with an ICER of $3513 per QALY. The graph of one-way sensitivity analysis can be found in the Additional file 1.

**Table 3:** Results of one-way sensitivity analyses of selected parameters

Parameters	Range	Preferred strategy*
Stage I		
Esophagectomy		
Complication**	0.119–0.35	EMR
Dead	0.018–0.111	EMR (ICER of $34,768 for esophagectomy at 0.041)
No recurrence to metastasis	0.002–0.0139	EMR (ICER of $24,377 for esophagectomy at 0.0109)
EMR		
Complication**	0.030–0.069	EMR
No recurrence to local recurrence	0.0076–0.0426	EMR (ICER of $532 for EMR vs EMR followed by ablation at 0.03389)
EMR followed by ablation		
Complication**	0.068–0.222	EMR
No recurrence to local recurrence	0.014–0.033	EMR
Utility of stage I	0.66–0.71	EMR
Utility stage II and III	0.74–0.78	EMR
Utility stage IV	0.73–0.77	EMR
Stage II and III		
Esophagectomy		
Complication**	0.25–0.39	CRT followed by surgery
Dead	0.0339–0.111	CRT followed by surgery
No recurrence to dead	0–0.0905	At 0.0452, ICER of $3513 for esophagectomy vs CRT followed by surgery
CRT followed by surgery		
Complication**	0.17–0.289	CRT followed by surgery (at 0.289, ICER of $2373)
Dead	0.04–0.105	CRT followed by surgery (at 0.105, ICER of $2493)
CRT		
Complication**	0.148–0.28	CRT followed by surgery
Dead	0.024–0.058	CRT followed by surgery
No recurrence to local recurrence	0.028–0.0528	CRT followed by surgery
No recurrence to metastasis	0.0221–0.0139	CRT followed by surgery
Utility stage II and III	0.74–0.78	CRT followed by surgery
Utility stage IV	0.73–0.77	CRT followed by surgery

^*^The sensitivity analysis is based on the QALY outcome, ICER reported per QALY

^**^Complications included; for esophagectomy: pulmonary infection, heart failure, anastomotic leakage, severe arrhythmia, bleeding, wound infection, atelectasis, and acute respiratory distress syndrome. For EMR: bleeding, perforation, prolonger hospitalization, stenosis, and pneumonia. For EMR followed by ablation: strictures, bleeding, pain, and perforation. For CRT followed by surgery: anastomotic leakage, peritonitis, mediastinitis, esophagotracheal fistula, wound complication, and cardiac complication. For CRT: anastomotic leakage, pneumonia, cardiac arrhythmia, chyle leak, wound infection, and thromboembolic event

**Fig. 2:**
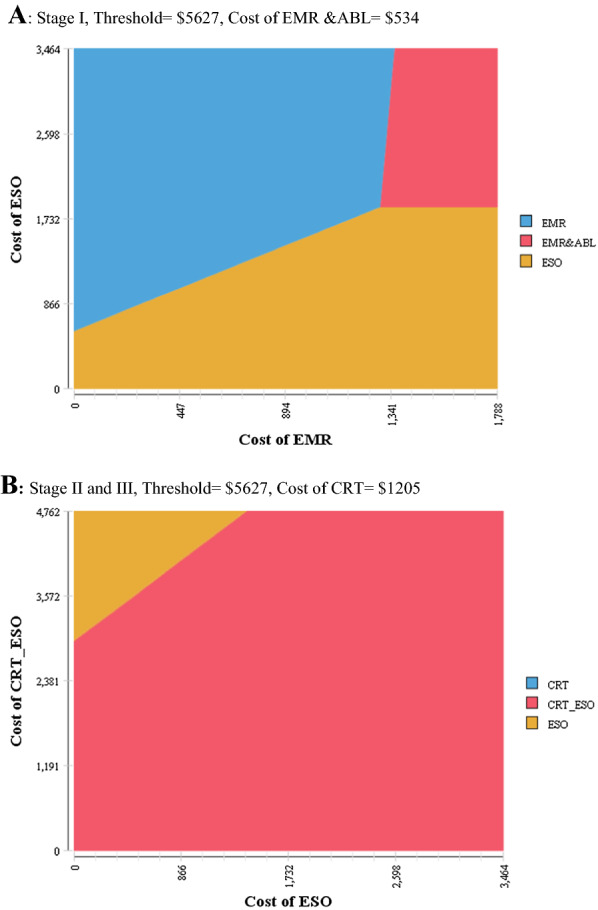
Results of sensitivity analysis for costs. *EMR*, endoscopic mucosal resection, *EMR* & *ABL*, endoscopic mucosal resection followed by ablation, *ESO*, esophagectomy, *CRT*, chemoradiotherapy, *CRT_ESO*, chemoradiotherapy followed by surgery

The sensitivity analysis of costs can be found in Fig. 2. Each area represents the cost-effectiveness of a particular intervention under specific costs. The esophagectomy intervention would not be a cost-effective strategy unless the cost of esophagectomy sharply decreased to less than $866 in stage I and less than $1232 in stages II and III.

## Discussion

This study aimed to evaluate the cost-effectiveness of treatment interventions for patients with ESCC according to the disease stage, using the Iranian health system (The clinical guidelines for treating patients with esophageal cancer in the Iranian health system have been developed based on NCCN guidelines). The EMR was a dominant intervention (lower cost and increased LYG and QALY) over esophagectomy and EMR followed by ablation in stage I. For patients with stages II and III ESCC, the CRT followed by surgery compared with CRT alone was cost-effective with an ICER of $2172 per QALY. CRT followed by surgery was also a dominant strategy over esophagectomy.

Our economic evaluation showed that the optimal treatment would be EMR in stage I of ESCC. A cost-saving of $1097 and $268 per patient was obtained for EMR treatment compared with esophagectomy and EMR followed by ablation, respectively. Furthermore, for patients with advanced ESCC (stage II and III), CRT followed by surgery saved $915 and 0.384 QALY compared to esophagectomy, and CRT followed by surgery was a cost-effective treatment with an ICER of $2172 per QALY versus CRT.

The number of studies associated with the economic evaluation of EC treatment is limited. However, studies have been conducted in recent years, evaluating the economics of EC intervention. Chu et al. developed a Markov model to evaluate treatment procedurals in the T1a and T1b of esophageal adenocarcinoma. They compared esophagectomy versus endoscopic treatment in terms of CEA. The results showed that esophagectomy in patients with T1a of EC resulted in more life-years gained than endoscopic therapy but lower QALYs compared to endoscopy. Also, they indicated that in patients with T1b, esophagectomy was not cost-effective compared to endoscopic treatment [22]. Khioe et al. reported that adjuvant statin therapy followed by surgery was dominant over no-statin therapy. This study reported a cost-saving of £6781 per patient [64]. Another study by Lin et al. evaluated the cost-effectiveness of neoadjuvant concurrent chemoradiotherapy (NCCRT) versus esophagectomy in locally advanced ESCC, using a payer’s perspective. They showed that NCCRT had higher costs and survival rates compared to esophagectomy. The ICER was estimated at US$ 39,060 per LYG [65].

The current study results were robust to variability and uncertainty using the Markov model’s estimates, as shown in Table 3. The sensitivity analysis indicated that changing individual parameters to the maximum and minimum levels did not change the base case results. For patients with intermediate ESCC, the probability of death and transition of no-recurrence state to metastasis in the esophagectomy strategy had a minor impact on the ICER. Esophagectomy could be cost-effective versus EMR, with an ICER of $34,768 per QALY, if the probability of mortality was reduced to less than 4% for the esophagectomy intervention. This ICER of $34,768 is more than three-times the Iranian GDP per capita. Most studies have reported a probability of more than 4% for postoperative mortality in esophagectomy [8, 66]. For patients with advanced ESCC, the base case results were only sensitive to the transition from no-recurrence to a dead state in esophagectomy. If this probability is reduced to less than 4%, compared to CRT followed by surgery, the esophagectomy intervention would be cost-effective with an ICER of $3513 per QALY. Furthermore, the cost sensitivity analysis showed that varying interventions cost did not change the base case results. If the cost of esophagectomy is reduced to more than 60%, the esophagectomy intervention would be cost-effective. At present, the Iran’s health system cannot reduce the cost of interventions to this level.

The current study has some limitations due to data availability and assumptions. First, we calculated the costs of treatment interventions based on the Iranian health system's perspective. Since health care systems in countries are different in terms of health services costs, generalizing the study findings is cautioned. Second, we extracted the Markov model data from different studies due to the lack of randomized controlled trials. These studies evaluated different patient populations with confounding variables that may have affected the results found herein. Third, the studies on the economic evaluation of cancer treatment interventions depended on the time of diagnosis. We assumed that patients would be diagnosed early. However, many patients were in the advanced stage when referred for treatment, especially in EC, due high mortality rates identified.

Despite the stated limitations, the study has several strengths. Most importantly, this study evaluated the cost-effectiveness of treatment strategies for ESCC patients based on stage. It can be a guide for therapists to determine the most cost-effective treatment for patients with ESCC. These results can reduce the treatment expenditure for patients and their families. This result is also relevant for countries with high EC rates, especially for low and middle-income countries.

## Conclusion

Based on available evidence, EMR appears to be the dominant strategy versus esophagectomy and EMR followed by ablation in the early stage. CRT followed by surgery is a cost-effective intervention compared to CRT alone and esophagectomy for patients with advanced ESCC. These results are sensitive to postoperative mortality, and the transition from no-recurrence to dead state on interventions. Since evidence-based policymaking for selecting the therapeutic producers depends on the analysis of clinical data and economic data to control the costs of cancers and resource allocation in the health sector, this study could provide insights to health care systems similar to Iran's.

## Supplementary Information

**Additional file 1**: **Appendix** **S1.** Results of one-way sensitivity analyses in Stage I. EMR: Endoscopic Mucosal Resection, EMR &ABL: Endoscopic Mucosal Resection followed by ablation, ESO: Esophagectomy. **Appendix** **S2.** Results of one-way sensitivity analyses in Stage II and III. CRT: Chemoradiotherapy, CRT_ESO: Chemoradiotherapy followed by surgery, ESO: Esophagectomy.

## Data Availability

The essential data is available in the article and we can provide upon request.

## Abbreviations

EC: Esophageal cancer
ESCC: Esophageal squamous cell carcinoma
CRT: Chemoradiotherapy
ICER: Incremental cost-effectiveness ratio
CEA: Cost-effectiveness analysis
ACER: Average cost-effectiveness ratio
WHO: World Health Organization
GDP: Gross domestic product
QALYs: Quality-adjusted life-years
NCCRT: Neoadjuvant concurrent chemoradiotherapy
