# Supplementary material for: A cost-effectiveness modeling study of treatment interventions for stage I to III esophageal squamous cell carcinoma
Source: Cost Eff Resour Alloc. 2022 Apr 2;20:16. doi: 10.1186/s12962-022-00352-5 (PMC8976992; doi:10.1186/s12962-022-00352-5)
Supplement: Supplementary file 1 — Additional file 1: Appendix S1. Results of one-way sensitivity analyses in Stage I. EMR: Endoscopic Mucosal Resection, EMR &ABL: Endoscopic Mucosal Resection followed by ablation, ESO: Esophagectomy. Appendix S2. Results of one-way sensitivity analyses in Stage II and III. CRT: Chemoradiotherapy, CRT_ESO: Chemoradiotherapy followed by surgery, ESO: Esophagectomy. [file 12962_2022_352_MOESM1_ESM.docx]

Appendix A: Results of one-way sensitivity analyses in Stage I. EMR: Endoscopic Mucosal Resection, EMR &ABL: Endoscopic Mucosal Resection followed by ablation, ESO: Esophagectomy.

| A: Probability of mortality for esophagectomy= 0.041  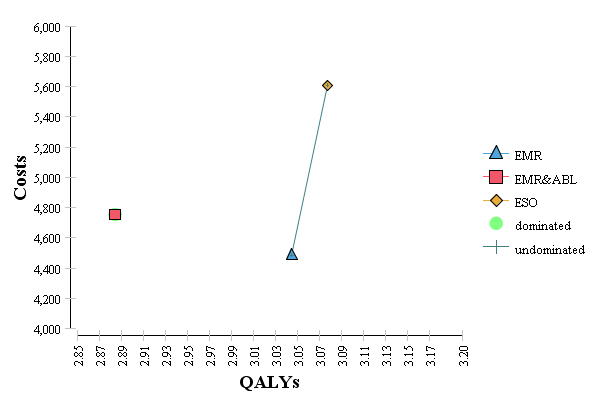 |
| --- |

| B: Probability of no-recurrence to metastasis for esophagectomy= 0.0109  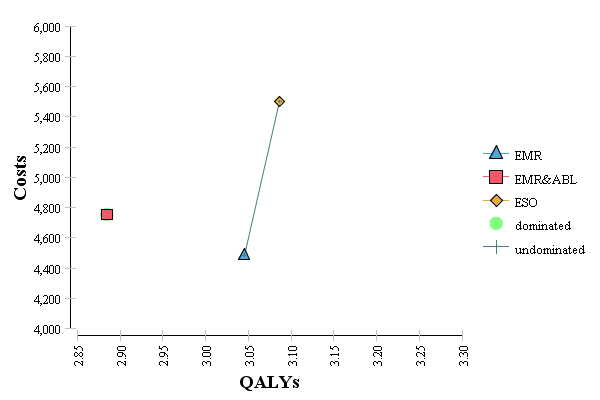 |
| --- |

| C: Probability of no-recurrence to local recurrence for EMR= 0.03389  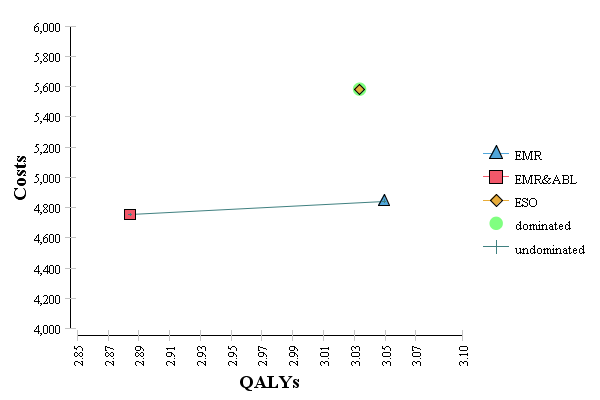 |
| --- |

**Appendix B:** Results of one-way sensitivity analyses in Stage II and III. CRT: Chemoradiotherapy, CRT_ESO: Chemoradiotherapy followed by surgery, ESO: Esophagectomy.

| A: Probability of no-recurrence to dead for esophagectomy= 0.0452  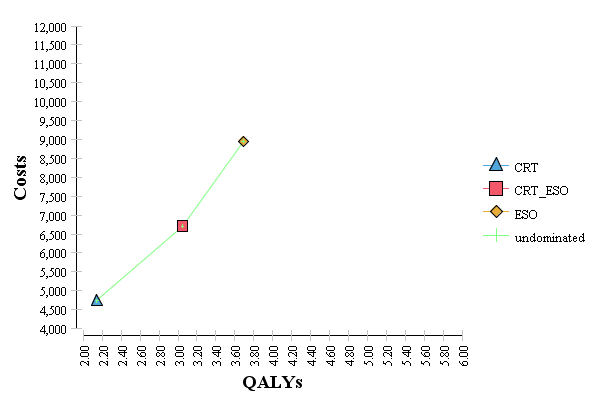 |
| --- |

| B: Probability of complication for CRT followed by surgery= 0.289  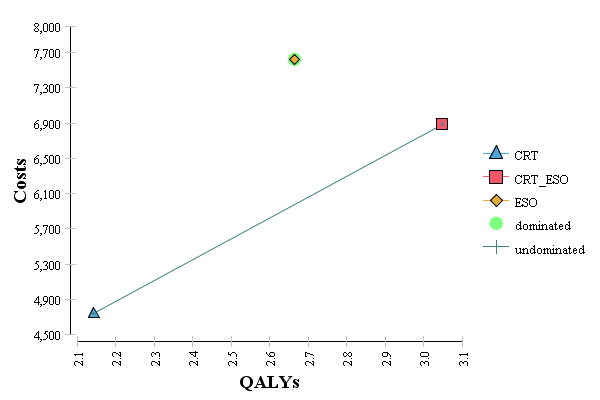 |
| --- |

| C: Probability of mortality for CRT followed by surgery= 0.105  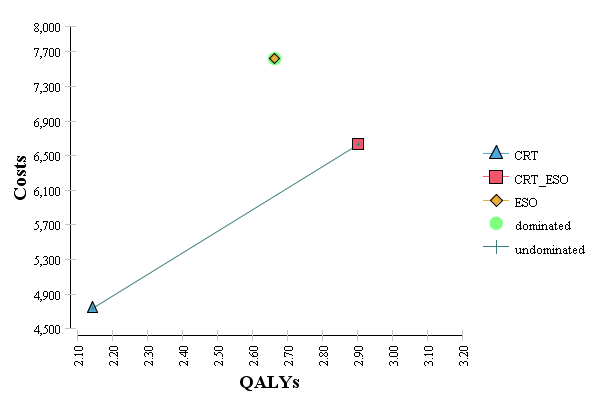 |
| --- |
